# Paclitaxel-related type I Kounis Syndrome in a very young patient with HER2-positive breast cancer and the role of genomics to disentangle a complex therapeutic scenario: a case report and narrative review

**DOI:** 10.1016/j.breast.2025.104465

**Published:** 2025-04-04

**Authors:** Javier Muñoz, Sabrina Nucera, Nuria Rubira Garcia, Isaac Cebrecos, Gabriela Oses, Sergi Ganau, Esther Sanfeliu, Pedro Jares, Mercedes Marín-Aguilera, Patricia Galván, Fara Brasó-Maristany, Olga Martínez-Sáez, Enric Cascos, Carme Font, Francesco Schettini

**Affiliations:** a Medical Oncology Department, Hospital Clinic of Barcelona, C. Villaroel 170, 08036, Barcelona, Spain; b Translational Genomics and Targeted Therapies in Solid Tumors Group, August Pi i Sunyer Biomedical Research Institute (IDIBAPS), C. Villaroel 170, 08036, Barcelona, Spain; c Department of Human Pathology "G. Barresi", University of Messina, 98131, Messina, Italy; d Allergy Service, Hospital Clinic of Barcelona, Barcelona, Spain; e Clinic Institute of Gynecology, Obstetrics and Neonatology, Hospital Clinic of Barcelona, Barcelona, Spain; f Faculty of Medicine, University of Barcelona, Barcelona, Spain; g Radiation Oncology Department, Hospital Clínic de Barcelona, Barcelona, Spain; h Department of Radiology, Diagnosis Imaging Center, Hospital Clinic of Barcelona, Barcelona, Spain; i Department of Pathology, Biomedical Diagnostic Center, Hospital Clinic of Barcelona, Barcelona, Spain; j Reveal Genomic, Barcelona, Spain; k Department of Cardiology, Hospital Clínic of Barcelona, Barcelona, Spain

**Keywords:** Kounis syndrome, HER2-Positive, Breast cancer, HER2DX, Genomics

## Abstract

We present the first documented oncologic case of a type I Kounis syndrome (KS) following paclitaxel administration, in a very young patient with HER2-positive(+) early-stage breast cancer (BC). KS is a relatively rare acute coronary syndrome triggered by anaphylactic or hypersensitivity reactions, of which there is limited awareness among healthcare providers. It is subdivided in four subtypes depending on cardiac artery medical history. While no established management guidelines exist, its treatment requires addressing severe infusion reactions while ensuring proper myocardial perfusion. We hereby illustrate its successful acute management and report on how tumor genomics through the novel HER2DX assay helped re-defining the entire neo/adjuvant oncologic strategy. HER2DX integrates tumor size and nodal involvement with 27 genes’ expression data tracking four biological BC-related and immunologic signatures so to estimate a prognostic and a predictive score. This report demonstrates how clinical and genomic data can be effectively integrated to optimize therapeutic decisions in HER2+ BC, offering a model for personalized care also in atypical and complex cases.

## Highlights

• This is the 1st reported case of a type I Kounis syndrome (KS) during paclitaxel administration in HER2+ breast cancer (BC).
• KS is a relatively rare acute coronary syndrome triggered by anaphylactic or hypersensitivity reactions.
• Our patient experienced an anaphylactic reaction and type I KS following the 1st neoadjuvant paclitaxel cycle.
• HER2DX assay integrates tumor size, nodal status and 27 genes to estimate a prognostic and a predictive score in HER2+ BC.
• HER2DX assay helped re-defining the entire neo/adjuvant oncologic strategy.

## 1 Introduction

HER2-positive (+) breast cancer is a heterogeneous disease [1], accounting for 10–20% of all breast tumors, with approximately half cases expressing also estrogen receptor (ER) and progesterone receptor (PgR) at immunohistochemistry (IHC) [2]. According to ASCO/CAP guidelines, HER2 positivity is defined as a complete and strong membrane staining resulting in a IHC score of 3+ in ≥10% of cancer cells, and/or amplification of the *ERBB2* gene detected with *in situ* hybridization (ISH) techniques with a HER2/CEP17 ratio cutoff of ≥2.0 and an average HER2 gene copy number ≥4.0 signals/cells [3]. In the context of early-stage disease, the HER2DX genomic assay was recently developed with the aim of supporting systemic treatment de-escalation or escalation. The test assesses the tumoral expression of 27 genes accounting for a luminal biology-related genomic signature, a proliferation-related signature, a HER2 amplicon signature and an immunologic signature tracking B cell response and immune activation. This genomic information is integrated with primary tumor size and nodal status to generates a risk score which translates in points estimations of the 10-year event-free survival in patients undergoing (neo)adjuvant trastuzumab-based chemotherapy, a predictive score accounting for the likelihood of achieving a pathologic complete response (pCR) in case of neoadjuvant trastuzumab-based chemotherapy and a standardized assessment of the *ERBB2* mRNA levels in the form of a third score [4]. The test is currently recommended in selected cases by the Spanish SEOM-GEICAM-SOLTI clinical guidelines (level of recommendation IIB) and acknowledged by the 18th St. Gallen international consensus guidelines for its practice-changing potential [5].

Kounis syndrome (KS), also known as allergic angina or allergic myocardial infarction, is a rare (annual incidence 0.02%) and potentially life-threatening clinical condition where an acute coronary syndrome, such as coronary spasm, acute myocardial infarction, and stent thrombosis, occurs in the setting of an allergic or hypersensitivity reaction [6]. Various triggers, including medications, environmental exposures, foods, and coronary stents, have been identified, and their number has increased in recent years [6]. Four types of this syndrome have been described in the literature, each characterized by different underlying mechanisms and clinical scenarios (Table 1) [6,7]. Type I KS occurs in patients with normal coronary arteries where an allergic reaction causes coronary artery spasm without any underlying coronary artery disease [6,7]. Its clinical management is challenging as it requires addressing the allergic reaction and the acute coronary syndrome simultaneously [8], and a multidisciplinary team is essential.

**Table 1:** Brief description of KS types and summary of associated oncologic cases reported in the literature.

TYPES OF KS	BRIEF DESCRIPTION				
*Type I*	Occurs in patients with normal coronary arteries where an allergic reaction causes coronary artery spasm without any underlying coronary artery disease [6,7]				
*Type II*	Involves patients with pre-existing atheromatous coronary artery disease. The allergic reaction can cause either coronary artery spasm or the rupture of an atheromatous plaque, leading to an acute coronary event [6,7]				
*Type III*	It is associated with patients who have had coronary stents implanted with a stent thrombosis due to hypersensitivity to the stent components or other allergens [6,7]				
*Type IV*	It is a newly proposed type that involves patients with a history of coronary artery bypass graft surgery [6,7]				

**CLINICAL CASES REPORTED IN LITERATURE**					
**Author (Year)**	**Chemotherapeutic agent**	**Cancer type**	**Age**	**Gender**	**Clinical/instrumental manifestation**

Gemici, G et al. (2007) [29]	Paclitaxel	Ovarian cancer	51	Female	ST-segment elevations in inferior and anterior leads Coronary vasospasm Acute coronary syndrome
Park, S et al. (2009) [30]	Paclitaxel	Ovarian cancer	63	Female	ST segment elevation in the V2-5 leads Coronary occlusion secondary to thrombus formation Acute myocardial infarction
Oneglia, C et al. (2011) [31]	Cisplatin/cyclophosphamide	Ovarian cancer	72	Female	ST elevation in the inferior leads Acute coronary vasopasm
Chang, PH et al. (2011) [32]	Oxaliplatin	Colorectal cancer	45	Male	ST elevation in leads II, III, and aVF Acute coronary vasospasm
Karabay, CY et al. (2011) [33]	5-fluorouracil	Cancer of unknown primary	49	Male	ST elevations in V5-V6 and DI-aVL leads Acute coronary vasospasm Acute myocardial infarction
Baroni, M et al. (2011) [34]	Carboplatin	Lung adenocarcinoma	50	Male	ST elevation in inferior leads (II, III, aVF) ST depression in V1-V3 Acute coronary vasospasm
Kido, K et al. (2014) [35]	Capecitabine	Colorectal cancer	47	Male	ST segment elevation on the lateral leads Ventricular fibrillation arrest
Esber, C et al. (2014) [36]	Paclitaxel	Triple negative breast cancer	47	Female	ST elevations in leads V1, V2 and V3 with reciprocal ST depression Acute coronary thrombus formation Acute myocardial infarction
Rawal, G et al. (2016) [37]	Paclitaxel	Esophageal cancer	63	Male	ST elevation in the inferior leads (lead II, III and aVF) Acute Inferior Wall Myocardial Infarction Acute coronary thrombus formation
Liang, HZ et al. (2021) [38]	Epirubicin	Bladder cancer	62	Female	ST in leads II, III and aVF ST depression in leads I, aVL, and V1–V5 Acute coronary vasospasm
Wang, B et al. (2021) [39]	Paclitaxel	Lung adenocarcinoma	57	Male	ST segment elevation with precordial lead reciprocal changes Acute coronary vasospasm
Giangrande, N et al. (2023) [40]	Oxaliplatin	Colorectal cancer	59	Female	ST depression in V3-V6 and D2-D3-AVF ST elevation in V1-AVL Acute coronary vasospasm
Puri, P et al. (2024) [41]	Carboplatin, paclitaxel and pembrolizumab/placebo	Endometrial adenocarcinoma	63	Female	ST elevation ATAK complex (Adrenaline, Takotsubo, Anaphylaxis, and KS) Acute coronary vasospasm

**Legend.** KS: Kounis Syndrome.

**Fig. 1:**
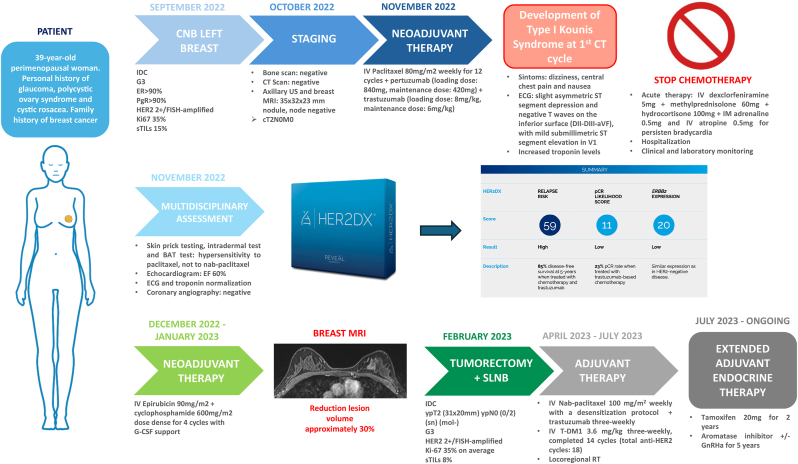
Case report timeline with clinicopathological and treatment details **Legend.** CNB: core needle biopsy; IDC: invasive ductal carcinoma; ER: estrogen receptor; PR: progesterone receptor; sTILs: stromal tumor-infiltrating lymphocytes; BAT: basophil activation test; G: grade; FISH: fluorescence *in situ* hybridization; CT: computed tomography; RT: radiotherapy; SLNB: sentinel lymph-node biopsy; MRI: magnetic resonance imaging; US: ultrasound; T-DM1: Trastuzumab emtansine; IV: intravenous; IM: intramuscular; sn: sentinel; mol-: molecularly negative; G-CSF: granulocytes colony stimulating factor; EF: ejection fraction; GnRHa: gonadotropin-releasing hormone analogue.

We hereby present the unique case of a non-smoker young patient without personal cardiovascular risk factors and no familiar history of ischemic myocardiopathy or dyslipidemia, affected by early-stage hormone receptor-positive (HR+)/HER2+ breast cancer who developed sudden hypotension and severe oppressive chest pain with electrocardiographic abnormalities and troponin elevation consistent with an acute coronary syndrome compatible with a type I KS, following paclitaxel administration (Fig. 1). No similar cases are reported in the literature in this scenario. We performed a literature review to identify similar cases, elucidate its frequency in Oncology, its pathophysiology and clinical management. We then focused on the HER2DX genomic assay's pivotal role in adapting the oncological management to the clinical complexity introduced by the KS (Fig. 1).

## 2 Case report

### 2.1 Clinical case presentation

**Fig. 2:**
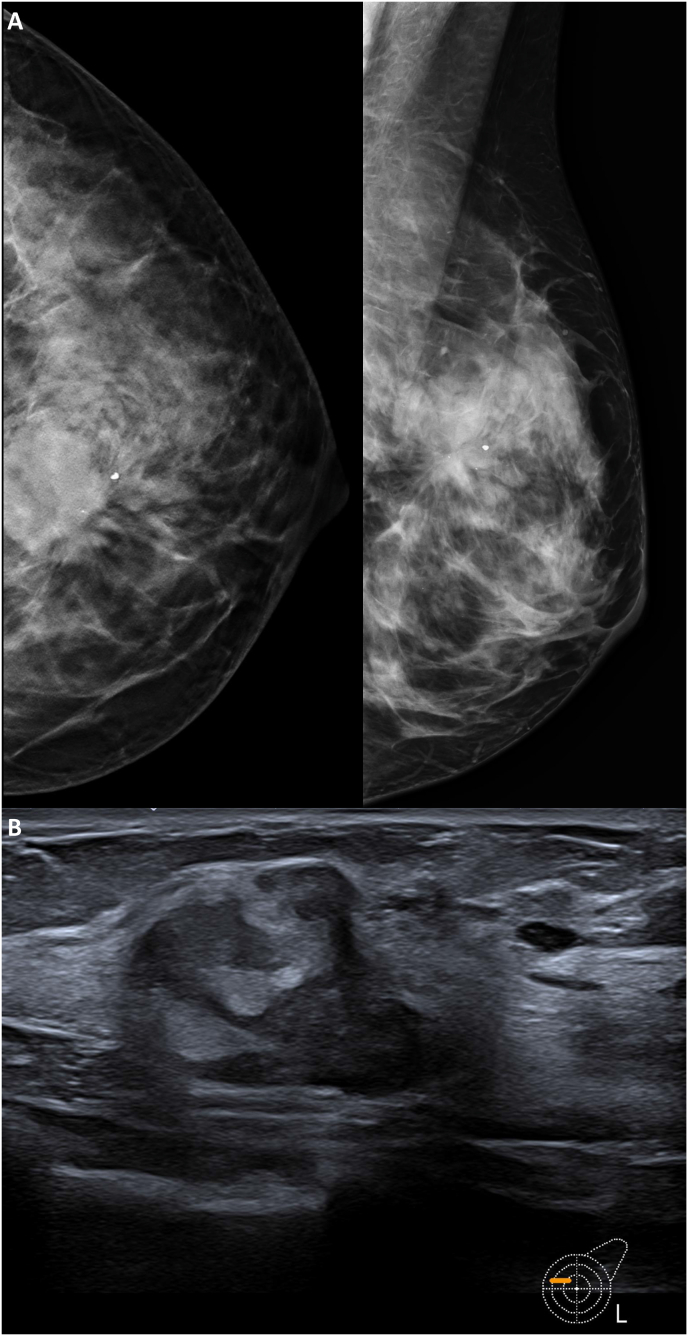
Radiology assessments obtained at diagnosis **Legend. A:** DBT of left breast (CC and MLO views) shows a irregular mass with spiculated margins and calcifications associated in the upper inner quadrant of left breast. **B:** Breast US shows a irregular and heterogeneous mass. DBT: digital breast tomography; CC: craniocaudal; MLO: mediolateral oblique; US: ultrasound.

**Fig. 3:**
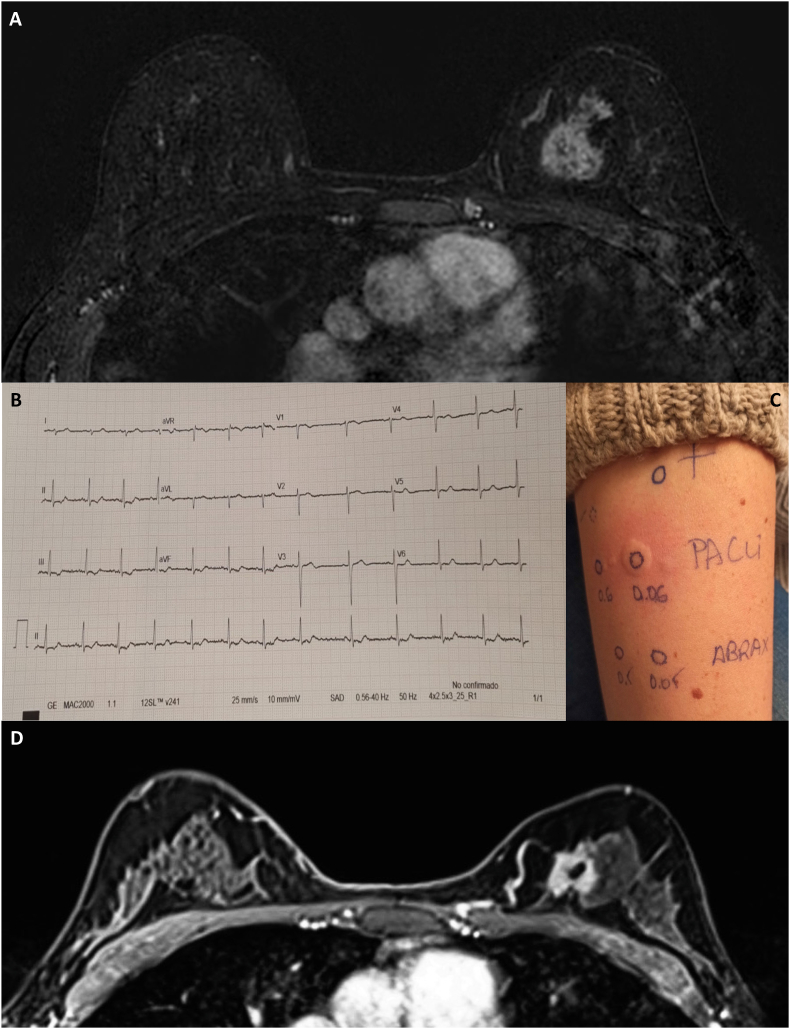
Patient's ECG obtained at the onset of central thoracic pain after paclitaxel infusion, intradermal reactions and MRI post neoadjuvant anthracyclines **Legend. A:** Pretreatment breast MRI showing a single left breast lesion (35 x 32 × 23 mm). **B:** The ECG displays a sinus rhythm at 82 bpm, with adequate PR and QRS segments, but with an asymmetric depression of the ST segment in the inferior leads (DII, DIII, aVF), with mild submillimetric ST segment elevation in V1. **C:** intradermal reaction to paclitaxel with concomitant negative test for nab-paclitaxel. **D:** Post-treatment breast MRI showing a reduction in lesion size of approximately 30 %. ECG: electrocardiogram; MRI: magnetic resonance imaging.

In September 2022, a 39-year-old perimenopausal woman with a personal history of glaucoma, polycystic ovary syndrome and cystic rosacea, following self-palpation, discovered a lump in her left breast. Subsequent digital breast tomosynthesis (DBT) revealed an irregular mass with spiculated margins and calcifications associated, highly suspicious for malignancy. Ultrasound (US) showed an irregular heterogeneous mass (approximately 3 cm) in the upper inner quadrant of the left breast (Fig. 2). A core needle biopsy revealed an invasive breast carcinoma of no special type (NST), grade 3, with Ki67 35%, stromal tumor-infiltrating lymphocytes (sTILs) 15%, ER expression >90%, PgR expression >90%, HER2 IHC score 2+, with *ERBB2* gene amplified according to a fluorescent *in situ* hybridization (FISH) for a HER2/CEP17 ratio of 2.1 and mean HER2/cell signal of 7.4 [3]. Breast cancer staging with magnetic resonance imaging (MRI) showed a single 3.5 cm lesion (Fig. 3A). Her family history of cancer included two maternal aunts diagnosed with breast cancer, a paternal uncle diagnosed with glioblastoma, and a maternal grandfather diagnosed with prostate adenocarcinoma. She reported menarche at the age of 13 with regular menstruation and no allergies to any medication. Axillary US, computed tomography (CT), and bone scintigraphy did not show axillary or distant metastases, resulting in a tumor AJCC stage IIA for cT2N0M0 [9]. A pre-treatment echocardiogram showed a non-dilated left ventricle with normal ejection fraction (60%), no valvular disease, and no pericardial effusion. Given the limited tumor extension, the patient was recommended neoadjuvant treatment with paclitaxel 80mg/m2 weekly for 12 cycles + pertuzumab (loading dose: 840 mg, maintenance dose: 420 mg) + trastuzumab (loading dose: 8 mg/kg, maintenance dose: 6 mg/kg) and further post-surgical systemic treatments guided by pathologic response. The patient received also genetic counselling and genomic testing, but no pathogenic germline variant in breast cancer-related genes was detected [10].

### 2.2 KS occurrence and acute management

On November 12, the patient initiated treatment with pertuzumab and trastuzumab at 12:00 p.m. which was completed without complications. At 1:20 p.m. paclitaxel infusion was started. After 5 min, the patient began experiencing dizziness, severe oppressive central chest pain, and nausea. Vital signs revealed arterial hypotension (70/40 mmHg), bradycardia (heart rate 40/minute), and adequate oxygen saturation (98% on room air). The infusion was immediately stopped. The patient underwent an electrocardiogram (ECG), which showed sinus rhythm with slight asymmetric ST segment depression and negative T waves on the inferior surface (DII-DIII-aVF), along with mild submillimetric ST segment elevation in V1 (Fig. 3B). Intravenous (IV) dexchlorpheniramine 5 mg, methylprednisolone 60 mg, hydrocortisone 100 mg and intramuscular adrenaline 0.5 mg were administered. IV atropine 0.5 mg was further administered due to persisting bradycardia. The acute treatment ultimately led to an ECG normalization. At 2:30 p.m., with persistent chest pain and hypotensive state, the patient was transferred to the Emergency Department. Blood tests showed elevated troponin I levels (106.2 ng/mL; reference interval <45.2 ng/mL). Cardiological evaluation diagnosed anaphylactic shock related to paclitaxel administration with a possible co-occurring type I KS. Subsequent monitoring of troponin levels showed an increase followed by a progressive decrease (902.3 > 2011.6>1933.1 > 1393.5>861.2 > 654.9 ng/mL). The patient underwent clinical monitoring, with resolution of chest pain and hypotensive state, and a new echocardiography showed preserved ejection fraction, without notable abnormalities. On November 13, the patient was discharged home from the hospital.

### 2.3 Redefinition of the oncologic management with the HER2DX assay

After careful multidisciplinary evaluation involving oncologists, allergists and cardio-oncologists, allergic tests were programmed and a HER2DX genomic assay was performed on the diagnostic biopsy [11]. Results showed a high risk of cancer recurrence (relapse risk score 59), with a low probability of pCR (pCR likelihood score 11) and low *ERBB2* expression (*ERBB2* mRNA level score 20), consistently with the previous IHC/FISH borderline result. This suggested a potentially limited response to anti-HER2 agents, the need for multiagent chemotherapy to improve outcomes and low margins for treatment de-escalation due to the molecular high risk of relapse, along with the young patient's age. On November 23, the patient underwent skin prick testing with paclitaxel (6 mg/mL) and nab-paclitaxel (5 mg/mL) and intradermal test with paclitaxel (0.6 and 0.06 mg/mL) and nab-paclitaxel (0.5 and 0.05 mg/mL). Positive reaction to paclitaxel 0.06 mg/mL was observed, without any reaction to nab-paclitaxel (Fig. 3C). Finally, the flow cytometry-based basophil activation test (BAT) resulted in a weak positivity to paclitaxel, with negative response to nab-paclitaxel. We thus hypothesized a potential allergy to cremophore, rather than paclitaxel itself, as this is a well-known issue related to standard paclitaxel but not nab-paclitaxel, which is an albumin-bound formulation of paclitaxel that do not use cremophore as solubilizer [12]. However, additional time was necessary to confirm its safety. Therefore, the neoadjuvant strategy was modified and an anthracycline-based regimen with epirubicin 90mg/m2 + cyclophosphamide 600mg/m2 dose dense for 4 cycles with granulocyte colony-stimulating factor (G-CSF) support was proposed. The patient was thoroughly informed and the genomic assay's result favored the acceptance of the novel strategy proposed. Before starting the treatment, a new echocardiographic examination was performed, showing an ejection fraction of 60%. Non-invasive coronary angiography revealed no coronary artery disease. Chemotherapy was administered between November 2022 and January 2023 and was well tolerated. The patient underwent a breast MRI, which showed a reduction in the lesion volume by approximately 30% (Fig. 3D), then underwent tumorectomy with sentinel lymph-node biopsy on February 2023. The definitive histological examination showed an invasive carcinoma of NST, ypT2 (31 × 20mm) ypN0 (0/2) (sn) (mol-), grade 3, TILs 8%, HER2 2+/ISH-amplified, heterogeneous Ki-67 (5%–50%), with an average of 35%. Allergic studies were repeated after surgery and replicated previous results. Unfortunately, the unavailability of cremophore extract for testing prevented us to confirm our original hypothesis. However, since allergy to nab-paclitaxel was ruled out, the patient received adjuvant nab-paclitaxel 100 mg/m^2^ weekly for 12 cycles with a desensitization protocol (i.e. drug administered over a series of very gradual dose increments such that the sum total dose equals the original target dose of the drug) preceded by metilprednisolone 60 mg + ebastine 20 mg or cetirizine 10 mg, plus trastuzumab three-weekly from April 2023 to July 2023. Subsequently, considering the absence of pCR, the patient underwent treatment with trastuzumab-emtansine (T-DM1) 3.6 mg/kg three-weekly, of which completed 14 cycles (total anti-HER2 cycles: 18) with good tolerance and without the appearance of relevant adverse events.

From July 2023 to August 2023, the patient received adjuvant breast radiotherapy in a hypofractionated scheme with a total dose of 40.05 Gy in 15 fractions, followed by radiotherapy to the tumor bed (boost) with a total dose of 13.35 Gy in 5 fractions. In July 2023 the patient started adjuvant hormonal therapy with tamoxifen 20 mg daily, as she refused to undergo ovarian function suppression at least for the first couple of years. In this regard, a family history of early menopausal onset and amenorrhea for several months already before starting chemotherapy suggested a possible early menopause. As such, after 2 years of tamoxifen a switch to an aromatase inhibitor to reach a total of 7 years of extended adjuvant therapy will be proposed [13], with or without ovarian function suppression, depending on her menopausal status. The patient has continued serial cardiological check-ups, including clinical examination and echocardiography, with no abnormalities detected.

## 3 Discussion

Here we report the first case in literature of a patient with HR+/HER2+ early-stage breast cancer experiencing a type I KS following paclitaxel infusion and the role of genomics to disentangle such a complex therapeutic scenario and re-define the oncological management.

### 3.1 Standard management of stage I-II HER2+ breast cancer

The standard therapeutic approach for stage I HER2+ disease, especially if the primary tumor is <1.0 cm, is represented by primary surgery followed by adjuvant paclitaxel plus 1 year of adjuvant trastuzumab. Starting from stage II, a neoadjuvant approach usually based on double anti-HER2 blockade with trastuzumab and pertuzumab plus chemotherapy is preferred, followed by the completion of 1 year of adjuvant trastuzumab in case of pCR is achieved, or T-DM1, in case of residual disease [[14], [15], [16], [17]]. In all stages, the presence/absence of HR is clinically important for guiding the prescription of endocrine therapy [14]. In the APT single arm phase II trial, excellent 10-year outcomes were reported for patients with stage I-IIA HER2+ breast cancer treated with de-escalated adjuvant regimen consisting of weekly paclitaxel and 1 year of trastuzumab; in the NEOSPHERE trial, the addition of pertuzumab to chemotherapy and trastuzumab significantly improved pCR rates, with pCR being a strong favorable prognostic factor for HER2+ breast cancer [[18], [19], [20]]. Moreover, in the DAPHNE trial, patients with stage II-III HER2+ breast cancer were treated with neoadjuvant paclitaxel plus double anti-HER2 blockade with trastuzumab and pertuzumab, obtaining a pCR rate of 56.7% and excellent long-term outcomes without further adjuvant chemotherapy in case of pCR achievement [21]. Thus, at a first approach, our patient with a stage IIA HR+/HER2+ tumor could have been reasonably treated with neoadjuvant paclitaxel plus anti-HER2 double blockade and posterior adjuvant trastuzumab or T-DM1 depending on pathologic response and endocrine therapy. However, the patient experienced a severe allergic reaction with development of type I KS, that prevented us from continuing with the same approach and required a careful reassessment.

### 3.2 KS syndrome: pathophysiology, diagnosis, treatment and evidence in breast cancer

KS represents a multifaceted and intricate multisystemic disorder demanding swift diagnosis and intervention. Its pathophysiology involves a complex interaction between the immune and cardiovascular systems, characterized by conditions related to mast cell activation, the release of inflammatory mediators, and platelet activation in the context of allergic reactions [22]. Its diagnosis hinges upon a comprehensive assessment of clinical signs and symptoms, complemented by laboratory analyses and additional diagnostic procedures [23]. Laboratory tests, particularly the measurement of tryptase, cardiac enzymes, immunoglobulins (IgE) and cardiac troponins are useful in diagnosing cardiac damage. ECG may show signs of alterations in cardiac electrical conduction, with the most common finding being an ST segment elevation in anterior and inferior leads, while echocardiography and angiography provide information on coronary anatomy and any abnormalities of the cardiac wall [24]. The differential diagnosis of acute chest pain in patients with cancer receiving chemotherapy includes: catheter-related complications (thrombosis, pneumothorax, chemotherapy extravasation), pulmonary embolism and Takotsubo syndrome among others [25,26]. The treatment is complex due to the delicate balance required between addressing cardiac manifestations and managing allergic reactions. Drugs used to treat cardiac dysfunction (i.e. vasodilators) may worsen allergies, while therapies for allergies (i.e. atropine, norepinephrine) could potentially exacerbate cardiac symptoms. Moreover, the specific type of syndrome developed requires tailored supportive therapy including parenteral saline infusion, antihistamine drugs, corticosteroids and/or vasoactive amines [27]. In the type I variant of KS, treatment typically involves intravenous corticosteroids and H1 and H2 antihistamines. Vasodilators such as calcium channel blockers and nitrates may also be administered to alleviate vasospasm [28]. Unfortunately, there are no established guidelines due to its rarity. Regarding its association with chemotherapy, we performed a literature search on Pubmed on September 2024 and identified 13 published cases of diagnosed KS following chemotherapy drug infusion [[29], [30], [31], [32], [33], [34], [35], [36], [37], [38], [39], [40], [41]]. Five of these cases were associated with paclitaxel administration, with only one patient being diagnosed with breast cancer [29,30,36,37,39] (Table 1). None of those was of type I. In our clinical case, timely diagnosis of type I KS and acute treatment led to the remission of symptoms without any lasting effects on cardiac function. However, the development of this syndrome required a multidisciplinary discussion among a team of specialists to evaluate the available therapeutic options.

### 3.3 Tumor genomics to disentangle a problematic therapeutic scenario

In the last decade, accumulating evidence has pointed out that HER2+ breast cancer is a molecularly heterogeneous disease [1], for which a one-size-fit-all approach is progressively becoming inviable. The HERD2X assays helps dissecting this biological heterogeneity and provides the clinician with additional information that complement standard clinicopathological features. In this regard, given the complexity of our clinical case, we decided to rely on this assay to further decide on how to proceed.

Our patient had a high genomic risk of relapse according to HER2DX, which translated in a 15% probability at 5 years and 28% at 10 years of experiencing a tumor relapse even if treated with trastuzumab-based chemotherapy. To note, a HER2DX sub-analysis of the APT trial identified a proportion of patients with stage I-IIA at higher risk of relapse, for which this regimen appeared to be suboptimal both in terms of relapse-free interval and overall survival [18]. Moreover, in our patient the low pCR likelihood score was associated only to a limited 11% probability of achieving pCR with anti-HER2-based chemotherapy. Villacampa et al. recently demonstrated that neither dual anti-HER2 blockade nor multiagent chemotherapy are able to increase pCR rates in case of a HER2DX pCR-low score, but also that HER2DX risk score is prognostic beyond pathologic responses [42]. Importantly, the HER2DX *ERBB2* low score suggested a suboptimal sensitivity to anti-HER2 agents. In fact, HER2DX-low *ERBB2* mRNA levels (10–25% HER2+ tumors) have been associated to scarce responses and worse long-term outcomes to T-DM1 and anti-HER2 double blockade in multiple early-stage and metastatic cohorts [[43], [44], [45], [46]].

### 3.4 Treatment justification and conclusions

Overall, taking into account the implications of the assay's result, the very young patient's age, a known poor prognostic factor for HR+ breast cancer [47], as well as the additional time required to further clarify the safety of taxanes, we revised the therapeutic strategy accordingly. Specifically, we opted for multiagent chemotherapy with anthracyclines instead of single-agent taxane to better tackle the high risk of relapse. We persisted with the neoadjuvant approach to still provide a timely management of potential micrometastases, gain time to complete allergy tests and try to reduce the invasiveness of the surgical intervention. Moreover, being high likely the presence of residual disease, we gave to our patient the opportunity to receive adjuvant T-DM1 instead of trastuzumab alone, in order to maximize adjuvant anti-HER2 benefit, provided the low *ERBB2* score. In fact, once assured that continuing chemotherapy with nab-paclitaxel would have been a feasible option, the patient resumed the standard taxane + trastuzumab regimen after surgery. Then received adjuvant T-DM1 to complete one year of the most effective anti-HER2 treatment currently available in the absence of pCR [48]. In this way, the patient received all the best available systemic treatment options for her case, without significant additional therapeutic delays, providing also that alternative strategies (e.g. pertuzumab in adjuvant setting or neratinib post adjuvant trastuzumab) were not available in our regional context. Moreover, the genomic assay reinforced the patient's confidence in her healthcare providers' therapeutic decisions despite a traumatic treatment-related adverse event, and improved the oncologist's confidence in pursuing an escalated therapeutic approach despite the delicate clinical scenario, as also demonstrated elsewhere [49,50]. The main limitation of this report is the impossibility to determine the ultimate efficacy of the oncologic treatments administered based on a single case experience and the short follow-up. However, this report is significant as it presents the first documented case of type I KS in an oncological patient after paclitaxel treatment, highlighting its successful acute management. It also demonstrates how integrating clinical and genomic data can optimize therapeutic decisions in HER2+ breast cancer, offering a model for personalized care in complex cases.

## CRediT authorship contribution statement

**Javier Muñoz:** Writing – review & editing, Writing – original draft, Investigation, Conceptualization. **Sabrina Nucera:** Writing – review & editing, Writing – original draft, Visualization, Data curation, Conceptualization. **Nuria Rubira Garcia:** Writing – review & editing, Methodology, Investigation. **Isaac Cebrecos:** Writing – review & editing, Investigation. **Gabriela Oses:** Writing – review & editing, Investigation. **Sergi Ganau:** Writing – review & editing, Investigation. **Esther Sanfeliu:** Writing – review & editing, Methodology, Investigation. **Pedro Jares:** Writing – review & editing, Methodology. **Mercedes Marín-Aguilera:** Writing – review & editing, Methodology, Investigation. **Patricia Galván:** Writing – review & editing, Investigation. **Fara Brasó-Maristany:** Writing – review & editing, Investigation. **Olga Martínez-Sáez:** Writing – review & editing, Investigation, Conceptualization. **Enric Cascos:** Writing – review & editing, Investigation. **Carme Font:** Writing – review & editing, Writing – original draft, Supervision. **Francesco Schettini:** Writing – review & editing, Writing – original draft, Visualization, Supervision, Methodology, Conceptualization.

## Ethics and informed consent

The patient provided an explicit consent to report her clinical case in anonymized form. Being a case report, no Ethic Committee approval was required as *per* national and local legislation.

## Funding

None.

## Declaration of competing interest

The authors declare the following financial interests/personal relationships which may be considered as potential competing interests: **F. Brasó-Maristany** reports patent application (PCT/EP2022/086493, PCT/EP2023/060810, EP23382703 and EP23383369) and being part-time employee of Reveal Genomics. **M. Marín-Aguilera** is a full-time employee of Reveal Genomics. **O. Martínez-Sáez** reports advisory/consulting fees from Reveal Genomics, Roche and Astrazeneca, lecture fees from Daiichi Sankyo, Novartis, Pfizer and Eisai and travel expenses from Gilead and Novartis. **F. Schettini** reports honoraria from Novartis, Gilead and Daiichy-Sankyo for educational events/materials, advisory fees from Pfizer and travel expenses from Novartis, Gilead and Daiichy-Sankyo. The other authors have nothing to declare.
